# Mitochondria and FOXO3: breath or die

**DOI:** 10.3389/fphys.2013.00147

**Published:** 2013-06-20

**Authors:** Judith Hagenbuchner, Michael J. Ausserlechner

**Affiliations:** ^1^Department of Pediatrics II, Medical University InnsbruckInnsbruck, Austria; ^2^Tyrolean Cancer Research InstituteInnsbruck, Austria; ^3^Department of Pediatrics I, Medical University InnsbruckInnsbruck, Austria

**Keywords:** forkhead transcription factor, Bcl2-rheostat, BH3-only proteins, mitochondrial respiration, mitochondrial fission

## Abstract

Forkhead box O (FOXO) transcription factors are regulators of cell-type specific apoptosis and cell cycle arrest but also control longevity and reactive oxygen species (ROS). ROS-control by FOXO is mediated by transcriptional activation of detoxifying enzymes such as Superoxide dismutase 2 (SOD2), Catalase or Sestrins or by the repression of mitochondrial respiratory chain proteins resulting in reduced mitochondrial activity. FOXO3 also regulates the adaptation to hypoxia by reducing mitochondrial mass and oxygen consumption during HIF-1α activation. In neuronal tumor cells, FOXO3 triggers ROS-accumulation as a consequence of transient mitochondrial outer membrane permeabilization, which is essential for FOXO3-induced apoptosis in these cells. Cellular ROS levels are affected by the FOXO-targets Bim, BclxL, and Survivin. All three proteins localize to mitochondria and affect mitochondrial membrane potential, respiration and cellular ROS levels. Bim-activation by FOXO3 causes mitochondrial depolarization resulting in a transitory decrease of respiration and ROS production. Survivin, on the other hand, actively changes mitochondrial architecture, respiration-efficacy and energy metabolism. This ability distinguishes Survivin from other anti-apoptotic proteins such as BclxL, which inhibits ROS by inactivating Bim but does not alter mitochondrial function. Importantly, FOXO3 simultaneously also activates ROS-detoxification via induction of SESN3. In this paper we discuss the hypothesis that the delicate balance between ROS-accumulation by Bim-triggered mitochondrial damage, mitochondrial architecture and ROS-detoxifying proteins determines cell fate. We provide evidence for a FOXO self-reactivating loop and for novel functions of FOXO3 in controlling mitochondrial respiration of neuronal cells, which further supports the current view that FOXO transcription factors are information-integrating sentinels of cellular stress and critical modulators of cell homeostasis.

## The family of forkhead box O transcription factors

The family of mammalian forkhead box O (FOXO) transcription factors consists of the four members FKHR/FOXO1, FKHRL1/FOXO3, AFX/FOXO4, and FOXO6 which regulate apoptosis and cell cycle, immune response, energy state, stress resistance, and longevity. Although all four mammalian FOXO transcription factors share the same DNA binding motives (Obsil and Obsilova, 2011) and seem to have overlapping functions, knock out animals for single FOXO family members show different defects (Arden, 2008). FOXO1 knockout mice die *in utero* due to defective vasculature (Hosaka et al., 2004), FOXO3 knockout mice suffer from organ inflammation resulting from defective development of regulatory T-cells (Harada et al., 2010; Kerdiles et al., 2010). In contrast FOXO4 and FOXO6 knockout mice present with mild phenotypes (Zhu et al., 2011; Salih et al., 2012). Conditional triple-knock-out in the adult mouse causes a relatively mild neoplastic phenotype, i.e., these mice develop hemangiomas and thymic lymphomas, which suggests that FOXO1, FOXO3, and FOXO4 are involved in the maintenance of the hematopoietic stem cell population and the regulation of endothelial cell homeostasis (Paik et al., 2007; Tothova et al., 2007). In cultured neuroblastoma cells the activation of FOXO3 triggers the intrinsic death pathway and induces programmed cell death via induction of the pro-apoptotic BH3-only proteins Bim and Noxa (Obexer et al., 2007). In addition FOXO3 represses the apoptosis-inhibitor protein Survivin (Guha et al., 2009; Obexer et al., 2009) and determines the sensitivity of neuroblastoma cells to DNA-damaging chemotherapeutic agents. More recently it was shown that beside its function as a tumor-suppressor FOXO3 might also promote cancer cell survival. FOXO3 induces detoxification and stress resistance thereby contributing to tumor stem cell renewal (Naka et al., 2010) and protection of cancer cells from eradication during chemotherapy (Hui et al., 2008) and hypoxia (Bakker et al., 2007).

In this article we will discuss the current knowledge on the involvement of FOXO transcription factors in the regulation of cellular homeostasis with specific emphasis on mitochondrial integrity, morphology and activity. In addition we present our hypothesis that FOXO3 controls a delicate balance between mitochondrial reactive oxygen species (ROS)-generation and ROS-preventing or detoxifying processes, which is critical for cell death decision in neuronal cells.

## Growth factor signaling inactivates FOXO transcription factors

**Figure 1:**
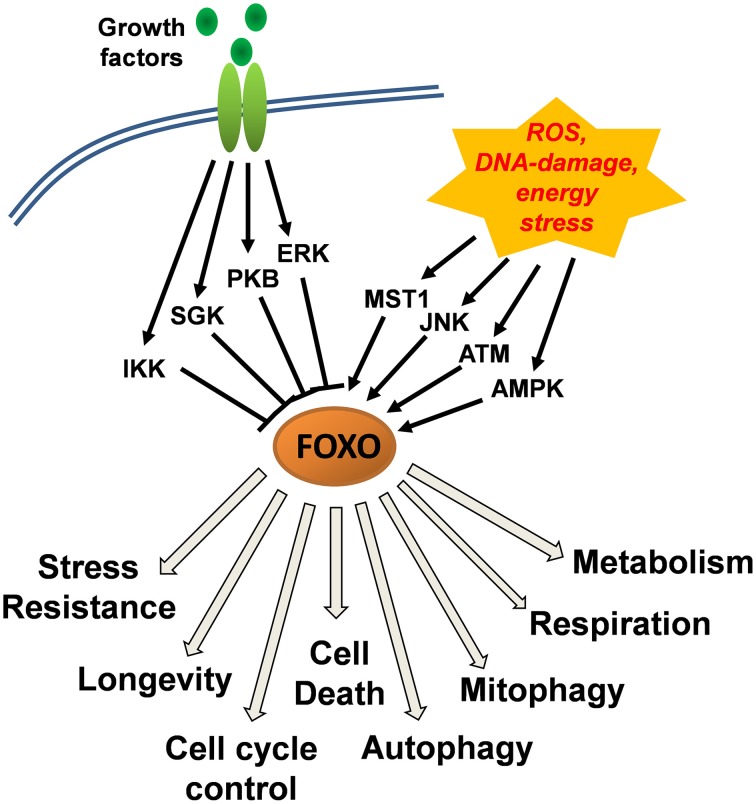
**Control of FOXO function downstream of growth-factor and stress-induced kinase signaling cascades**. Growth factor signaling via protein kinase B (PKB), serum- and glucocorticoid-induced kinase (SGK), IkB-kinase (IKK), and extracellular-signal regulated kinase (ERK) causes inactivation of FOXO transcription factors and their export from the nucleus. Jun-N-terminal kinase (JNK) and mammalian STE20-like protein kinase 1 (MST1) that are activated upon cellular stress, induce the nuclear accumulation of FOXOs. A main trigger of JNK activation is accumulation of cellular reactive oxygen species (ROS) which together with DNA-damage also activate ataxia telangiectasia mutated (ATM) kinase and induce the interaction between ATM and FOXO and its nuclear accumulation. AMP-activated kinase (AMPK) functions as a cellular energy sensor that increases the activity of nuclear FOXO factors. AMPK thereby acts in concert with either low growth factor availability or high cellular stress and further steers FOXO target recognition.

The activity and subcellular localization of FOXO transcription factors (except FOXO6) is regulated by various upstream regulators that modify FOXO proteins via phosphorylation, acetylation, methylation and mono/poly-ubiquitination (Eijkelenboom and Burgering, 2013). Phosphorylation at conserved serine/threonine residues by protein kinase B (PKB) and by serum- and glucocorticoid-induced kinase (SGK) induces the association of FOXO transcription factors with 14-3-3 proteins and their nuclear export and inactivation (Tzivion et al., 2011). This evolutionary conserved FOXO-inactivating pathway directly links FOXO activity to insulin and insulin-like growth factor signaling, suggesting that under normal growth conditions, FOXOs are inactivated and dispensable for the survival of cells. Similar to PKB and SGK, IkB-kinase (IKK) and extracellular-signal regulated kinase (ERK) phosphorylate distinct serines (Ser644 of FOXO3 by IKK and Ser294, Ser344, Ser425 of FOXO3 by ERK) thereby causing FOXO-inactivation (Figure 1). Importantly, phosphorylation by PKB, IKK, and ERK also constitutes a signal for poly-ubiquitination and proteasomal degradation of FOXO transcription factors thereby, in addition to functional inactivation, also reducing protein steady state levels (Matsuzaki et al., 2003; Hu et al., 2004; Yang et al., 2008).

## Stress kinase cascades that activate FOXO in response to ROS and DNA-damage

First evidence for the participation of FOXO transcription factors in ROS regulation was found in *C.elegans* studying the FOXO3 homologue DAF-16. There, DAF-16 regulates a stress-resistant-state, where metabolism is shut down and the worm enters so called Dauer-formation to extend lifespan during nutrient deprivation (Braeckman and Vanfleteren, 2007). FOXO3 is also thought to participate in longevity regulation by detoxification of ROS. Several groups found the mitochondrial enzyme superoxide dismutase 2 (SOD2) (Kops et al., 2002), the peroxisome-located Catalase (CAT) (Tan et al., 2008), the antioxidant enzyme peroxiredoxin III (Chiribau et al., 2008) or the redox enzyme sestrin3 (SESN3) (Chen et al., 2010; Hagenbuchner et al., 2012a) induced by FOXO in different tissues. Also the growth arrest and DNA damage repair enzyme GADD45a is activated after stress signaling by FOXO transcription factors and contributes to the survival of damaged cells and stress resistance (Furukawa-Hibi et al., 2002).

Cellular stress induced by accumulation of ROS or DNA-damage overrides the growth factor-induced functional inactivation of FOXO. The response to ROS involves Jun-N-terminal kinase (JNK) and mammalian STE20-like protein kinase 1 (MST1) which phosphorylate FOXO transcription factors directly (Essers et al., 2004; Lehtinen et al., 2006; Sunters et al., 2006) or also target its binding partner 14-3-3 protein (Sunayama et al., 2005). The direct phosphorylation by JNK or MST1 induces the release of FOXO3 from 14-3-3 proteins and causes nuclear accumulation. However, as phosphorylation occurs at a conserved serine in the forkhead domain (Ser209 of FOXO3) it has not been completely clarified, how this phosphorylation affects consensus sequence recognition by FOXO3. According to crystal structures and band shift analyses (Brent et al., 2008), DNA binding by FOXO1 is reduced or event abrogated suggesting that during activation by JNK or MST1, FOXO might at least in part interact with other transcription factors such as p53 (Chung et al., 2012) or c-Myc (Ferber et al., 2012) and thereby regulate target gene expression.

An important sensor of DNA-damage response is the ataxia telangiectasia mutated (ATM), a member of the phosphoinositol-3-kinase-like kinase family. ATM coordinates together with ATM-related kinase (ATR) the cellular response to DNA-damage by activating DNA-repair and signaling pathways. ATM senses DNA double strand breaks and is also activated by ROS in mammalian cells (reviewed in Ditch and Paull, 2012). ATM interacts with FOXO3 and p53 during DNA-damage (Tsai et al., 2008; Chung et al., 2012) and ROS-response (Yalcin et al., 2008) suggesting that these proteins are tightly interconnected during stress signaling. We recently provided evidence that the FOXO3-ATM complex also overcomes epigenetic silencing of the caspase-8 gene in human neuroblastoma cells by activating the ATM downstream target CREB which in turn triggers methylation-independent activation of the caspase-8 promoter (Geiger et al., 2012).

AMP-activated kinase (AMPK) is a sensor of cellular energy homeostasis and is activated by high AMP to ATP ratios. AMPK phosphorylates FOXO3 on at least six different serine/threonine residues (Thr179, Ser399, Ser413, Ser439, Ser555, Ser588, and Ser626), which does not change subcellular localization of FOXO3 but increases its transcriptional activity and may modulate differential promoter recognition (Greer et al., 2007). AMPK only phosphorylates nuclear FOXO3 thereby acting in concert with growth factor withdrawal or cellular stress signals. The exact mechanism of FOXO3 transcriptional activation by AMPK has not been completely elucidated but phosphorylation by AMPK increases the interaction with CREB-binding protein (CBP) (Wang et al., 2012) and p300 which both affect FOXO3 transcriptional activity and promoter recognition by acetylation. Phosphorylation of FOXO by AMPK has been linked to FOXO-induced autophagy (Chiacchiera and Simone, 2009), neuronal cell death (Davila et al., 2012), and muscle atrophy (Sanchez et al., 2012). Importantly, fission of mitochondria triggers AMPK activation and FOXO3-induced autophagy, which removes mitochondria and contributes to muscle atrophy (Romanello et al., 2010). This suggests an energy-sensing network between AMPK, FOXO3 and mitochondrial architecture.

## Steering FOXO function by acetylation, methylation and interaction with other transcription factors

Besides kinase cascades FOXO transcription factors are subject to additional post-translational modifications such as acetylation/deacetylation processes, most prominent via acetylation of lysines in the forkhead domain by CBP/p300 (Wang et al., 2012) or deacetylation by the protein deacetylase sirtuin-1 (SIRT1) (Brunet et al., 2004; Kobayashi et al., 2005). Whereas acetylation enhances the expression of pro-apoptotic FOXO-targets, SIRT1 modulates the transcriptional function of FOXO3 in a way which inhibits FOXO3-induced expression of pro-apoptotic genes and increases the expression of genes involved in cell-cycle regulation, DNA-repair and stress resistance. FOXO-deacetylation by SIRT1 may therefore also contribute to longevity and survival of tumor cells, questioning the general view of FOXOs as tumor suppressor proteins (reviewed in (Calnan and Brunet, 2008). Another posttranslational modification is methylation by protein arginine methyltransferases (PRMT) 1 and 6 that add methyl groups to arginine on substrate proteins. In the case of FOXO transcription factors, PRMT1 targets arginine Arg248 and Arg250 (of FOXO1) within the PKB consensus motive, which prevents PKB-mediated phosphorylation of Ser256 (FOXO1). As a consequence nuclear export in presence of active PKB is inhibited and the transcriptional activity of FOXO transcription factors is increased (Yamagata et al., 2008). In contrast SET-domain containing protein 7 (SETD7/SET9), a lysine-methyltransferase was shown to methylate Lys270 in FOXO3, which inhibits DNA-binding, induction of FOXO3 target genes such as Bim and neuronal apoptosis (Xie et al., 2012). Therefore protein methylation significantly steers the activity of FOXO transcription factors and also affects their posttranslational modification by protein kinases.

Furthermore, FOXO proteins have been shown to cooperate with cofactors such as Smad3/4, p53, as well as with nuclear androgen-, glucocorticoid- and retinoic acid receptors (reviewed in, Calnan and Brunet, 2008; van der Vos and Coffer, 2008). Recently it was also shown that FOXO3 interacts with β-catenin and that this interaction converts the transcriptional activity of FOXO3 to promote metastasis instead of apoptosis in colon cancer (Tenbaum et al., 2012).

A recent report suggests that FOXOs may also directly measure the redox status in a cell via reversible oxidation/reduction of cysteine. Oxidation of cysteines in FOXO proteins causes the covalent binding of p300 and CBP via disulfide bonds. These modifications directly affect the transcriptional activity of FOXO transcription factors and thereby allow them to act as sensors of cellular redox status (Dansen et al., 2009). Together with phosphorylation by oxidative stress induced kinases such as JNK or MST1, this mechanism may directly affect target gene regulation by FOXO transcription factors and modulate the cellular response to ROS.

## FOXO3 as a trigger for mitochondrial ROS

**Figure 2:**
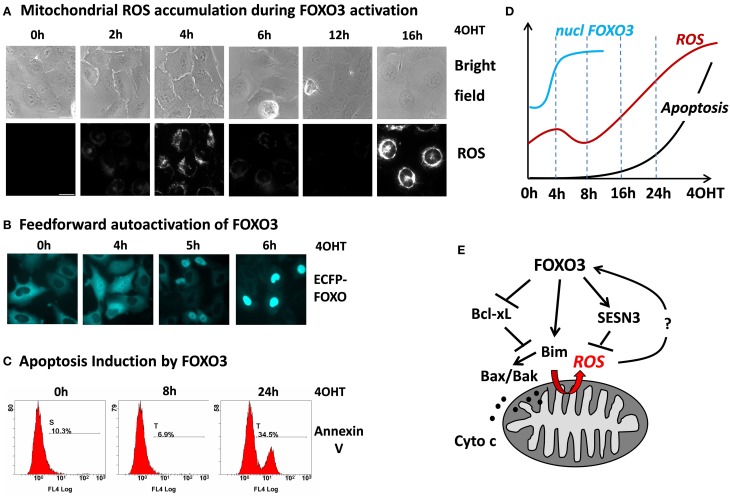
**FOXO3 activation causes biphasic ROS accumulation at mitochondria leading to feed forward activation and apoptosis**. SH-EP neuroblastoma cells expressing a conditional FOXO3-ERtm protein were treated with 4-OH-tamoxifene (4OHT), which leads to functional activation of this transgene. FOXO3 induces a first ROS accumulation at 4 h with complete SESN3-dependent decay of ROS between 8 and 14 h and strong secondary accumulation of ROS afterwards that finally leads to apoptotic cell death. Accumulation of ROS was detected using MitoTracker Red CMH_2_XROS (Invitrogen) by live-cell imaging in an Axiovert200M microscope (Zeiss) **(A)**. Subsequent to the first ROS peak triggered by FOXO3(A3)ERtm nuclear accumulation of ECFP-FOXO3wt is observed suggesting amplifying feed forward activation of additional FOXO3 molecules after primary FOXO3 activation **(B)**. Apoptosis was determined by Annexin-V staining in a FC500 flow cytometer (Beckman-Coulter) **(C)**. The chronology of cellular events in response to FOXO3 activation: after 4 h ECFP-tagged FOXO3 starts to accumulate in the nucleus (blue line, data based on quantification of nuclear FOXO3 by live cell imaging analysis), ROS accumulates first after 4 h, followed by decay to background ROS levels and a second much more intensive ROS accumulation (red line) that also marks the onset of cell death (black line, based on time course experiments using flow cytometric analysis of annexin-V staining). The data was compiled from analyses shown in **(A–C)**, additional unpublished data or data from (Hagenbuchner et al., 2012a) **(D)**. Model of cell death/stress resistance regulation by FOXO3 involving the biphasic accumulation of mitochondrial ROS downstream of Bim, which may constitute a feed forward signal to overcome the ROS-protective capacity of SESN3 **(E)**.

Although FOXO3 has been shown to induce a number of genes that protect against ROS suggesting that they play a critical role in keeping cellular ROS low, we recently demonstrated that in primary neurons and neuroblastoma cells FOXO3 may also increase mitochondrial ROS levels (Hagenbuchner et al., 2012a). The accumulation of ROS was essential for FOXO3-induced cell death in these cell types, since co-treatment with the ROS inhibitor N-Acetyl-L-cystein (NAC) rescued neuroblastoma cells from FOXO3-induced apoptosis. This uncovers an interesting possible feedback regulation between ROS and FOXO3 at mitochondria: on one hand FOXO3 is activated in response to elevated cellular ROS levels and on the other hand FOXO3 itself triggers ROS accumulation by interrupting mitochondrial outer membrane integrity. To investigate such a possible self-amplifying regulation we infected SH-EP/FOXO3 cells that carry a conditional 4OHT-activated FOXO3ERtm fusion protein with a retrovirus coding for a wild-type ECFP-FOXO3 fusion protein. This ECFP-FOXO3 fusion protein can be used to monitor FOXO3 nuclear accumulation by live cell fluorescence microscopy in response to various stimuli (Obexer et al., 2009; Geiger et al., 2012; Hagenbuchner et al., 2012a,b). In SH-EP neuroblastoma cells the PI3K-PKB signaling pathway is highly active, which causes cytoplasmic retention of FOXO3 (Obexer et al., 2007, 2009). In these cells, we studied whether activation of the conditional, PKB-independent FOXO3 allele and FOXO3-induced increase of mitochondrial ROS constitutes a signal to activate cellular FOXO3 protein despite active PKB. A first ROS accumulation was observed already 4 h after FOXO3-activation and at this time point ECFP-FOXO3wt is already equally distributed between nucleus and cytoplasm of the majority of the cells. Interestingly at 6 h, when the first ROS wave already declines, wild-type ECFP-FOXO3 accumulates in the nucleus of the neuroblastoma cells (Figure 2). This supports the hypothesis that some active FOXO3 molecules within a cell may trigger also the nuclear accumulation and thereby hyper-activation of other FOXO3 molecules. The primary, partial activation of FOXO increases the levels of pro-apoptotic Bim, which in turn causes damage to mitochondria, partial release of Cytochrome-*c* and increased ROS production (Hagenbuchner et al., 2012a). The secondary FOXO3 activation might at least in part be regulated by the first ROS accumulation and explain why some FOXO3 targets are immediately activated, such as Bim, whereas others are induced (such as p27^Kip1^, data not shown) or repressed (such as BclxL), (Hagenbuchner et al., 2012a) in delay. Although the relevance of ROS in this FOXO3-induced FOXO3 activation has to be proven, these observations support the hypothesis that oxidative stress activates FOXO3, which enhances further mitochondrial ROS and that this feedback-loop causes an avalanche-like, secondary activation of additional FOXO3 molecules leading to the second wave of ROS accumulation and apoptotic cell death (Figure 2).

## The connection between FOXO, pro-apoptotic Bcl2 proteins, respiration and ROS

When analyzing the events that lead to the first accumulation of ROS at the mitochondria, we identified the pro-apoptotic FOXO3-target Bim as a trigger of transitory mitochondrial outer membrane permeabilization. Bim is rapidly induced (within 2–8 h) by FOXO3 (Obexer et al., 2007), leading to translocation of Bax and to a first minor release of Cytrochrome-*c*, which correlates with the first ROS accumulation (Hagenbuchner et al., 2012a). Knock-down of Bim rescues neuroblastoma cells from ROS-accumulation and FOXO3-induced apoptosis, whereas tetracycline-regulated Bim-expression alone is sufficient to induce ROS. These observations imply that FOXO3 interrupts mitochondrial function by transient outer membrane permeabilization via Bim, which results in Bax/Bak activation and in leaking out of the electron-acceptor Cytochrome-*c* (Hagenbuchner et al., 2012a). As a result of inefficient electron transfer ROS accumulate at mitochondria. The amount of released Cytochrome-*c* seems not sufficient in these neuronal cells to induce immediate apoptosome-mediated caspase-9 activation and cell death, as first signs of apoptosis are evident much later after 18–24 h. Whereas the knock-down of Bim prevents FOXO3-induced ROS accumulation and cell death induction shRNA-mediated elimination of Noxa/PMAIP1, another BH3-only protein that is directly regulated by FOXO3 in neuronal cells, does not influence FOXO3-induced ROS accumulation. This suggests that a weak BH3-only protein such as Noxa, which cannot induce apoptosis by itself but acts as apoptosis-sensitizer is also not potent enough to cause ROS accumulation at mitochondria. The hypothesis that mitochondrial apoptosis is directly connected to transitory permeabilization of the outer mitochondrial membrane leading to mitochondrial ROS accumulation is further supported by the Bim-neutralizing pro-survival protein BclxL: if overexpressed BclxL not only prevents FOXO3-induced cell death but also the biphasic ROS accumulation thereby directly linking FOXO3-induced ROS to the balance of pro- and antiapoptotic Bcl2 proteins at mitochondria (Figures 2, 4). Simultaneously with Bim induction and ROS accumulation, mitochondrial respiration drops to about 70% of untreated controls, but recovers again to 99% after decay of the first ROS wave (Hagenbuchner et al., 2012a). Consistent with the hypothesis that FOXO3-induced mitochondrial ROS results from the detrimental effects of Bim on mitochondrial function, Bim overexpression causes a significant drop of respiration that coincides with high levels of ROS. On the other hand the overexpression of BclxL, which is repressed by FOXO3, also efficiently preserves respiration during FOXO3-activation (Hagenbuchner et al., 2012a). This suggests that FOXO3-activation by e.g., oxidative stress causes an imbalance of FOXO3-regulated Bcl2 proteins in neuronal cells leading to partial membrane permeabilization, transitory decrease in respiration and accumulation of ROS at mitochondria.

In contrast to these “acute FOXO3 effects” on mitochondria in neuronal cells additional mechanisms have been discovered how FOXO3 regulates mitochondrial mass, respiration and ROS production. Two groups recently demonstrated that FOXO3 is activated downstream of hypoxia-inducible factor-1α (HIF-1α) during hypoxia and is involved in the repression of nuclear-encoded mitochondrial genes during hypoxia. In this case, FOXO3 controls the adaptation to low oxygen and slowly shuts down mitochondrial activity by antagonizing c-Myc function. Hypoxia usually increases ROS production from mitochondria, which feeds back into stabilization of HIF-1α. Under hypoxic conditions, FOXO3 prevents hypoxia-induced ROS and HIF-1α stabilization by ROS, which contributes to tumor growth in xenograft models (Jensen et al., 2011; Ferber et al., 2012).

A third mechanism by which FOXO3 modulates mitochondrial activity is by directly regulating mitochondria-encoded genes. In response to glucose restriction in fibroblasts and skeletal muscle cells FOXO3 is phosphorylated by AMPK and subsequently imported into mitochondria where it forms a protein complex containing FOXO3, SIRT3, and mitochondrial RNA polymerase (mtRNAPol). This complex activates the expression of mitochondria-encoded genes, increases mitochondrial respiration and contributes to muscle adaptation during nutrient restriction (Peserico et al., 2013). How this complex affects mitochondrial structure has not been investigated yet.

## FOXO3-induced mitochondrial ROS—a balance between life and death?

So does FOXO3-induced early ROS accumulation already define the non-reversible activation of programmed cell death? Interestingly, after the first peak around 4 h after FOXO3 activation mitochondrial ROS almost completely goes back to baseline (Figure 2A and red line in schematic presentation), which suggests that in parallel to mitochondrial damage also rescue pathways are activated. In a large number of different cell types FOXO-transcription factors play a critical role in the detoxification of ROS and several FOXO3-targets were described for this cell-protective effect, among them SOD2, Catalase (Kops et al., 2002; Hasegawa et al., 2008; Tan et al., 2008) peroxiredoxin III (Chiribau et al., 2008) or the redox enzyme sestrin3 (SESN3) (Chen et al., 2010). SOD2 and Catalase are critically involved in the detoxification of superoxide-radicals and peroxide in various cell types and their activity might explain the decay of ROS and recovery of mitochondrial respiration after the first ROS peak during FOXO3 activation. However, SOD2 and Catalase were not regulated in neuroblastoma cells and therefore do not seem to contribute to the fluctuations of ROS levels during FOXO3-induced apoptosis (Hagenbuchner et al., 2012a). Neuronal cells rely on the thiol-reducing system based on thioredoxin and glutathione, which act as reducers of cellular peroxides (Budanov et al., 2004). In Microarray analyses, however, we identified the antioxidant enzyme SESN3 as a FOXO3 target in neuronal and neuroblastoma cells. SESN3 has two different functions: it acts as an antioxidant factor and is critical for the regeneration of peroxiredoxins. On the other hand SESN3 is also an inhibitor of target of rapamycin complex 1 (TORC1), which may affect FOXO3 activation via the PKB pathway (Budanov and Karin, 2008; Chen et al., 2010). When SESN3 induction is abrogated by short-hairpin technology this also prevents the transitory decline in ROS during FOXO3 activation and accelerates FOXO3-induced apoptosis in neuronal cells. This suggests that FOXO3 activate both, ROS production via Bim-induced mitochondrial damage and in parallel a ROS detoxifying pathway via SESN3. SESN3 induction and ROS detoxification therefore represent a rescue pathway for neuronal cells after FOXO3 activation. The balance between ROS-inducing and ROS-detoxifying molecular players determines whether the cell undergoes FOXO3-induced apoptosis or not.

The second ROS accumulation observed after about 16 h (Figure 2) is associated with phosphorylation of p66/SHC, a splice variant of the growth factor SHC, which localizes to mitochondria (Hagenbuchner et al., 2012b). Phosphorylation of p66/SHC correlates with increased H_2_O_2_ production (Giorgio et al., 2005), suggesting that prolonged FOXO3 activation overcomes the protective effect of SESN3 and continuous oxidative stress leads to phosphorylation of p66. Increased ROS finally causes oxidation of the Cytochrome-*c*-binding protein cardiolipin, which results in elevated levels of free Cytochrome-*c* in the inter-membrane space and finally to effective release of Cytochrome-*c* from mitochondria and apoptosis induction via apoptosome formation and caspase activation (reviewed in, Huttemann et al., 2011).

## Mitochondrial reorganization and effects of FOXO3

Mitochondrial activity and connectivity regulates oxidative phosphorylation and thereby intracellular ATP production. Electrons from energy rich NADH and FADH2 are transferred through the chain of four large enzyme complexes, leading to a proton flux via the inner mitochondrial membrane. As a side product, mitochondrial respiration leads to production of ROS, mainly through complex I, III and the reduced ubiquinol pool. ROS from complex I are mainly released on the matrix side of mitochondria, whereas complex III releases ROS into the matrix and the inter-membrane space. Within the inter-membrane space free Cytochrome-*c* acts as natural ROS detoxifier by removing unpaired electrons from superoxide leading to O_2_ formation and by conversion of hydrogen peroxide. During apoptosis induction, ATP production is reduced and mitochondria undergo reorganization to release Cytochrome-*c* into the cytosol which leads to a sharp increase of ROS from complex I (reviewed in, Sena and Chandel, 2012).

The mitochondrial shape and size are controlled by members of the fusion/fission family which, together with the Bcl2 family, control mitochondrial fragmentation and Cytochrome-*c* release (Karbowski et al., 2006; Sheridan et al., 2008). The main mammalian fission protein is the GTPase dynamin-related protein 1 (DNM1L/Drp1), whose activity is mainly regulated by phosphorylation at Ser616 and Ser637. Ser616 phosphorylation by Cdk1/cyclinB affects GTPase effector domain (GED) function and leads to translocation of Drp1 to the mitochondria during mitosis. De-phosphorylation on Ser637 is thought to be mediated by calcineurin which facilitates the translocation of Drp1 to distinct foci at the outer membrane of mitochondria, where oligomerization of Drp1 induces the fragmentation of mitochondrial networks into single mitochondria (reviewed in, Elgass et al., 2013). Other regulators of mitochondrial fusion, mitofusin 1 and 2 (MFN1 and MFN2) and optic atrophy1 (OPA1) also regulate mitochondrial fusion dynamics. MFN1 and MFN2 are located at the mitochondrial outer membrane and are responsible for connecting separate mitochondria. OPA1 is localized inside the intermembrane space and cooperates with MFN1 and MFN2 to fuse the inner and outer membranes of different mitochondria (reviewed in, Elgass et al., 2013).

Drp1, MFN1, and MFN2 were shown to cooperate with distinct members of the Bcl2 family in the reorganization of mitochondria during apoptosis induction. Active Drp1 facilitates Bax oligomerization during outer membrane permeabilization (Karbowski et al., 2006). BclxL and Bcl2 can bind MFN2, but not MFN1 and BclxL also interacts with Drp1, which interestingly increases its GTPase activity and leads to apoptosis-independent fragmentation of mitochondria in specific cell types (Delivani et al., 2006; Sheridan et al., 2008). Therefore, BclxL can promote either fusion or fission, even in the same cell type.

So how does FOXO3 affect mitochondrial architecture in neuronal cells? We observed de-phosphorylation of Drp1 at Ser637 and its translocation to the mitochondria 8 h after FOXO3 activation, suggesting that Bim-induced ROS may lead to cellular stress-induced de-phosphorylation of Drp1 (Hagenbuchner et al., 2012b). At the same time, Bax is recruited to the mitochondria and small amounts of Cytochrome-*c* are released to the cytoplasm. After 24 h FOXO3 induces MFN1 and MFN2 expression, which correlates with the onset of apoptosis and reorganization of mitochondria. Ectopic expression of BclxL not only blocked Cytochrome-*c* release and apoptosis by sequestering Bim but also prevented the induction of the fusion/fission machinery proteins by FOXO3 (Hagenbuchner et al., 2012b). This implies that induction of Bim and repression of BclxL by FOXO3 significantly affect the mitochondrial fusion/fission machinery via Drp1 recruitment, which, in addition to ROS accumulation and BH3-only-induced Bax-oligomerization may contribute to FOXO3-induced Cytochrome-*c* release and apoptosis (Hagenbuchner et al., 2012b). Beyond Bcl2-family members FOXO3 affects mitochondrial activity and shape also via regulating the FOXO3 target Survivin.

## Survivin uncouples mitochondrial fission from cytochrome-*c* release and apoptosis induction

**Figure 3:**
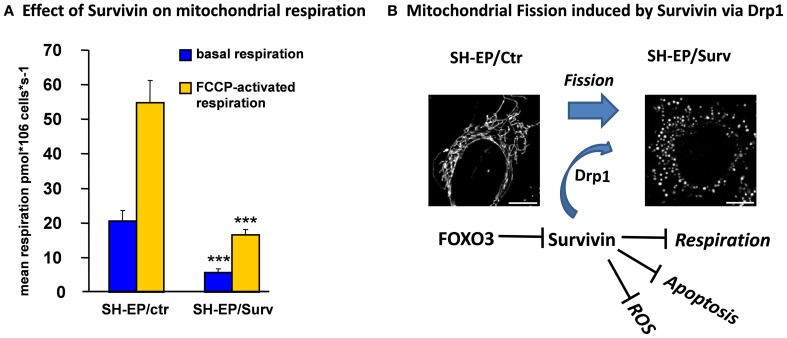
**The FOXO3-target Survivin reduces mitochondrial respiration and induces mitochondrial fission in neuronal tumor cells via Drp1 recruitment to mitochondria**. Ectopic expression of the anti-apoptotic protein Survivin in neuroblastoma cells reduces basal and FCCP-activated mitochondrial respiration to about one third of control cells. Oxygen consumption of the cells and mitochondrial function were analyzed by high-resolution respirometry (Kuznetsov et al., 2008), using a two-channel Oroboros Oxygraph respirometer. This reduced respiratory activity results from almost absence of respiratory complex I activity (data not shown; ^***^P < 0.001, student's *t*-test) **(A)**. Survivin recruits DNM1L/Drp1 to mitochondria and induces mitochondrial fission. Microscopic images were acquired on an Axiovert200M microscope equipped with an Apotome_2 module **(B)**. Mitochondrial fission is associated with apoptosis-protection and significantly reduced capability to accumulate ROS in response to FOXO3 activation or treatment with chemotherapeutic agents (see text) (Hagenbuchner et al., 2012b).

Survivin belongs to the family of Inhibitors of Apoptosis Proteins (IAPs) and was reported to inhibit apoptosis only when located to the mitochondria (Dohi et al., 2007; Obexer et al., 2009). Survivin contains the characteristic zinc-binding BIR domain but lacks the typical RING domain of other IAP family members. Apoptosis inhibition was ascribed to the ability of Survivin to directly bind and stabilize XIAP, which directly interferes with caspase activation (Dohi et al., 2004). These mechanisms suggest that Survivin prevents apoptosis downstream of mitochondria. However, in neuroblastoma cells we observed a different mode of action of Survivin: Survivin mRNA expression is rapidly repressed by FOXO3 and this leads to rapid loss of cytoplasmic Survivin, whereas mitochondrial Survivin shows significantly higher stability. Ectopically expressed Survivin reduces the number of CMXRos-negative cells during FOXO3-activation, suggesting a role for Survivin at the level of mitochondria (Obexer et al., 2009). In concordance we observed that Survivin prevents Bax accumulation at the mitochondria and the release of Cytochrome-*c* into the cytoplasm after FOXO3-activation. Interestingly, Survivin interferes with FOXO3-induced ROS accumulation. Similar to knock-down of Bim or ectopic expression of BclxL, Survivin inhibits FOXO3-induced ROS, Bax activation and Cytochrome-*c* release (Hagenbuchner et al., 2012b). In contrast to BclxL, which prevents Drp1 accumulation at mitochondria, Survivin-overexpressing cells showed increased Drp1 levels but reduced Drp1 phosphorylation (unpublished data) and therefore significantly higher amounts of mitochondria-associated Drp1 than control cells (Hagenbuchner et al., 2012b). These increased Drp1 levels resulted in fragmented mitochondria in Survivin-expressing cells (Figure 3). The mitochondrial fission phenotype is reversibly either by the use of the Drp1-inhibitor Mdivi-1 or by knock-down of Drp1 with short-hairpin technology, which both lead to mitochondrial fusion. Also the reduction of Survivin levels reverses the mitochondrial phenotype, suggesting that Survivin actively contributes to mitochondrial fission via Drp1 recruitment. In contrast to the current dogma that Drp1 translocation to mitochondria and mitochondrial fission is associated with apoptosis induction the Survivin-induced fission even protects cells against FOXO3-induced apoptosis and confers resistance to chemotherapeutic agents (Hagenbuchner et al., 2012b). This protective effect is associated with efficient inhibition of FOXO3-induced ROS, which may be ascribed at least in part to significant reduction of mitochondrial respiration by Survivin. In neuronal tumor cells moderate overexpression of Survivin lowers endogenous respiration to about one third of control cells via inactivation of respiratory complex I (Figure 3). Survivin-induced changes of mitochondrial shape and mitochondrial respiration reprograms cellular energy metabolism so that the cells become dependent on glycolysis and sensitive to glycolysis inhibitors (Hagenbuchner et al., 2012b). Thereby, by affecting cellular Survivin expression FOXO3 affects mitochondrial shape, respiratory activity and energy metabolism.

**Figure 4:**
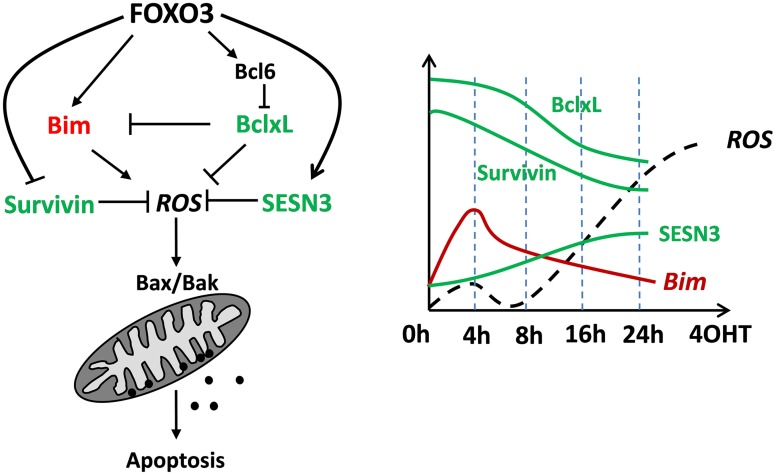
**A conceptional view on how FOXO3 regulates mitochondrial ROS and apoptotic cell death in neuronal cells and neuroblastoma tumor cells**. Transitory induction of pro-apoptotic Bim by FOXO3 constitutes a first mitochondria-damaging signal that triggers the primary ROS peak at 4 h. In parallel the ROS-detoxifying SESN3 accumulates and critically regulates decay of the ROS after 4–6 h. Bim-neutralizing BclxL and Survivin are both repressed by FOXO3, thereby lowering the ability of the cell to cope with apoptosis-inducing signals. This concerted regulation of pro- and anti-apoptotic ROS-affecting proteins overcomes the protective effect of SESN3 after about 16 h leading to a sharp and continuous increase in ROS, which finally leads to apoptotic cell death. The schematic presentation is based on several time-course immunoblot experiments and data from fluorescence-based mitochondrial ROS measurements not included in this paper.

FOXO3 was originally described as a tumor-suppressor protein that activates cell death inducers such as TRAIL, Fas ligand, Bim or Noxa or controls detoxification of ROS. In the meantime this view has been challenged as it was shown that FOXO3 may also control mitochondrial respiration during adaptation to hypoxia or is even imported into mitochondria to regulate expression of mitochondrial genes. The fact that FOXOs are inactive under optimal growth conditions suggests that these transcription factors function as homeostasis regulators during stress rather than as typical tumor suppressors. According to our recent studies FOXO3 induces mitochondrial ROS as an essential second messenger during Bcl2-protein controlled apoptotic cell death but also detoxifying proteins that counteract ROS accumulation (Figure 4). Importantly, FOXO3 also represses the proto-oncogene Survivin, which we discovered to regulate the mitochondrial fusion/fission machinery and mitochondrial respiration by interfering with complex I activity. By this, the FOXO3-target Survivin reprograms energy metabolism and activates glycolysis to produce energy for survival. So FOXO3 is a transcription factor that directly or indirectly affects mitochondrial respiration, ROS accumulation and even mitochondrial shape, which on one hand affects apoptosis sensitivity of tumor cells, but also may have significant impact on life-span in multicellular organisms.

### Conflict of interest statement

The authors declare that the research was conducted in the absence of any commercial or financial relationships that could be construed as a potential conflict of interest.
